# Exploring Elinor Ostrom's principles for collaborative group working within a user-led project: lessons from a collaboration between researchers and a user-led organisation

**DOI:** 10.1186/s40900-024-00548-4

**Published:** 2024-01-29

**Authors:** Bella Wheeler, Oli Williams, Becki Meakin, Eleni Chambers, Peter Beresford, Sarah O’Brien, Glenn Robert

**Affiliations:** 1 Nuffield Department of Primary Health Care Sciences, University of Oxford, Radcliffe Primary Care Building, Radcliffe Observatory Quarter, Woodstock Road, Oxford, OX2 6GG UK; 2 Florence Nightingale Faculty of Nursing, Midwifery & Palliative Care, King’s College London, 57 Waterloo Road, Waterloo, SE1 8WA UK; 3 Shaping Our Lives National User Network CIC, 30 St Giles’, Oxford, OX1 3LE UK; 4 School of Allied Health Professions, Nursing and Midwifery, The University of Sheffield, Barber House Annexe, 3a Clarkehouse Road, Sheffield, S10 2LA UK; 5 School of Health Sciences, University of East Anglia, Research Park, Norwich, NR4 7TJ UK

**Keywords:** Collaborative group working, User-led organisation, Inclusion, Inclusive involvement, Methodology, Innovation, Ostrom

## Abstract

**Background:**

Some research has been undertaken into the mechanisms that shape successful participatory approaches in the context of efforts to improve health and social care. However, greater attention needs to be directed to how partnerships between researchers and user-led organisations (ULOs) might best be formed, practiced, managed, and assessed. We explored whether political economist Elinor Ostrom’s Nobel prize winning analysis of common pool resource management—specifically eight principles to enhance collaborative group working as derived from her empirical research—could be usefully applied within a user-led project aiming to co-design new services to support more inclusive involvement of Disabled people in decision-making processes in policy and practice.

**Methods:**

Participant observation and participatory methods over a 16-month period comprising observational notes of online user-led meetings (26 h), online study team meetings (20 h), online Joint Interpretive Forum meetings (8 h), and semi-structured one-to-one interviews with project participants (44 h) at two time points (months 6 and 10).

**Results:**

Initially it proved difficult to establish working practices informed by Ostrom’s principles for collaborative group working within the user-led project. Several attempts were made to put a structure in place that met the needs of both the research study and the aims of the user-led project, but this was not straightforward. An important shift saw a move away from directly applying the principles to the working practices of the group and instead applying them to specific tasks the group were undertaking. This was a helpful realisation which enabled the principles to become—for most but not all participants—a useful facilitation device in the latter stages of the project. Eventually we applied the principles in a way that was useful and enabled collaboration between researchers and a ULO (albeit in unexpected ways).

**Conclusions:**

Our joint reflections emphasise the importance of being reflexive and responsive when seeking to apply theories of collaboration (the principles) within user-led work. At an early stage, it is important to agree shared definitions and understanding of what ‘user-led’ means in practice. It is crucial to actively adapt and translate the principles in ways that make them more accessible and applicable within groups where prior knowledge of their origins is both unlikely and unnecessary.

## Abstract (plain language summary)

Academic researchers and members of Shaping Our Lives—a national network and user-led organisation of Disabled people and service users—came together to explore whether Elinor Ostrom’s Nobel Prize winning work on collaborative group working could usefully inform efforts to co-design new services to promote the inclusive involvement of Disabled people in decision-making processes. We wanted to see if Ostrom’s ‘principles for collaborative group working’ were relevant to and could perhaps facilitate a co-design process led by a user led organisation. At first, we struggled to decide how Ostrom’s principles might inform the user-led project. We tried different ways to achieve this and eventually found an approach that most but not all of us found helpful. An important change we made was to stop focusing on how the members of the user-led group were collaborating together and instead to apply the principles to specific aspects of the co-design project that were complex and could be responded to in multiple ways. By the end of the 16-month study we had found a way of using the principles to better enable collaboration between academic researchers and a user-led organisation (although not in the way we had initially anticipated). We learned how Ostrom’s principles could be used to facilitate discussion of aspects of project work that are complex and the pros and cons of different plans of action. This project has demonstrated that collaboration between researchers and user-led organisations can be challenging but also has great potential for shared learning.

## Background

Contemporary research environments ought to be informed by, respond to the needs of, and foster collaborations with communities and citizens [1]. While the theory and practice of participatory research has progressed through various mandatory requirements in the field of health services research [2–6], recent evidence suggests research methodologies have not evolved sufficiently to ensure users and other citizens regularly and meaningfully contribute to research agendas, design, and practice [7–15]. This has led to two inter-related methodological priorities: firstly, the generation and piloting of novel methods to better support the translation of participatory theory into practice and secondly, feasibility studies conducted jointly by researchers and public and community members and groups (e.g., members of the public, patients, service users, user-led organisations, etc.) to explore any benefits and challenges of using these methods.

In response, we conducted a 16-month feasibility study as part of a partnership between social science researchers and Shaping Our Lives, a national user-led organisation specialising in the inclusive involvement of Disabled people and people marginalised due to other and intersecting social and personal characteristics. The collaboration sought to explore whether political economist Elinor Ostrom’s Nobel prize winning analysis of common pool resource management—specifically her eight principles for collaborative group working [16, 17]—could be usefully applied in the context of Shaping Our Lives’ aim to co-design new services to support more inclusive involvement of Disabled people in decision-making processes in policy and practice.

Ostrom’s ‘Governing the Commons’ found that certain conditions facilitate groups of people to sustainably manage common pool resources (CPR) [17]. CPR are defined as consisting of a natural or human-made resource system, where it is costly (but not impossible) to exclude potential beneficiaries from obtaining benefits from its use e.g., irrigation systems, forests, and fisheries. Without such management these resources are susceptible to ill-use with detrimental social outcomes, e.g., over-fishing and deforestation. Based on her analysis of a global database of case studies, Ostrom distilled a set of eight design principles the presence or absence of which largely explained the how effectively groups collaborated to manage CPRs [17] (Table 1):

**Table 1:** Ostrom’s core design principles

1. Clearly defined boundaries	The identity of the group and the boundaries of the shared resource are clearly delineated
2. Proportional equivalence between benefits and costs	Members of the group must negotiate a system that rewards members for their contributions. High status or other disproportionate benefits must be earned. Unfair inequality poisons collective efforts
3.Collective-choice arrangements	Group members must be able to create at least some of their own rules and make their decisions by consensus. People do not generally like to be told what to do but will work for mutually agreed group goals
4. Collective endeavours are inherently vulnerable to ‘free-riding’ and active exploitation	Collaborative efforts are unlikely to be successful unless there are strategies for norm-abiding members of the group to detect and manage such activities without great cost to themselves
5. Graduated sanctions	Transgressions need not require heavy-handed punishment, at least initially. Often gossip or a gentle reminder is sufficient, but more severe sanctions must also be ‘waiting in the wings’ for use when necessary
6. Conflict resolution mechanisms	It must be possible to resolve conflicts quickly and in ways that are perceived as fair by members of the group
7. Minimal recognition of rights to organize	Groups must have the authority to conduct their own affairs. Externally imposed rules violate principle 3
8. For groups that are part of larger social systems, there must be appropriate coordination among relevant groups	Every sphere of activity has an optimal scale. Any collective should recognise and form appropriate relationships with other groups within the system they operate. These relationships should not undermine the autonomy of the group to make collective decisions but should recognise that working effectively within a system requires some degree of cooperation between groups

A subsequent evaluation of 91 CPR case studies by Cox et al. in 2010 found the principles remained well supported empirically [18]. A later Ostrom collaboration highlighted the potential benefit of applying the design principles to understanding and supporting the efficacy of any collaborative group work, not just the management of CPRs; the authors encouraged others to use the principles as ‘a practical guide for improving the efficacy of groups in real-world settings’ [17].

To date, however, Ostrom’s design principles have been under-utilised. Despite the identification of their universal potential, the principles have predominantly been retrospectively applied to evaluate the management of CPRs [17]. One notable and very recent exception in the contemporary healthcare context applied Ostrom's work to the development amongst organisations of shared rules governing the utilisation of limited local resources [19]. However, this was not a participatory research project and the principles have never been tested within partnerships between health and social care researchers and service users.

Our research team recently took up this challenge by framing participatory research as a collaborative group activity to which Ostrom’s design principles can be applied. We retrospectively applied this methodology to a case study in Los Angeles, USA, where citizens returning to the community from jail co-designed priorities for local system-change and service improvement with a team of representatives from local African-American church groups and researchers [20]. We found some principles were applied instinctively within this co-design project (without prior knowledge of them) while others were not. Non-adherence with the absent principles appeared to limit the efficacy of the collaboration, suggesting that explicitly engaging with, adapting, and applying Ostrom’s principles from the beginning of future participatory research endeavours could be beneficial. Having applied this methodological innovation retrospectively with promising results we wanted to adapt, apply, and evaluate it prospectively in partnership with a user-led organisation.

To do this a partnership was formed between academic researchers at King’s College London and members of Shaping Our Lives—a national network and user-led organisation of Disabled people and service users. Shaping Our Lives successfully applied for funding from the UK National Lottery to co-design new services to support more inclusive involvement of Disabled people in decision-making processes in policy and practice, in a project titled the Inclusive Involvement Movement (IIM). We wanted to explore together whether Ostrom’s ‘principles for collaborative group working’ were relevant to and could perhaps facilitate the IIM project. More specifically, our research questions focused on whether a methodological innovation derived from Ostrom's analysis could (a) enhance Shaping Our Lives’ project to co-design services and (b) facilitate participatory research practice between researchers and a user-led organisation.

## Methods

We adopted participant observation and participatory methods [21, 22] throughout the 16 months and conducted semi-structured one-to-one interviews at two timepoints with project participants. Rather than ‘delicately lurking’ [23] and occupying a solely observational role, an academic researcher was initially positioned to work *with* the IIM project group in the adaptation and implementation of the principles. The IIM project group comprised seven members drawn from the broader Shaping Our Lives membership group and one member of staff. As we discuss below, it took significant time to establish our ways of working. Eventually the project consisted of three groups:

- the IIM group responsible for co-designing services to support the increased inclusion of Disabled people in decision-making processes in policy and practice
- a study group (comprising the academic researchers and two representatives from Shaping Our Lives—the chair of the organisation and the head of projects) exploring the application of the principles within the IIM project
- Joint Interpretive Forums [24] as a space for both groups to come together to reflect upon and discuss the work of the IIM in reference to Ostrom’s principles.

BW (academic researcher), BM (Shaping Our Lives head of projects), and PB (Chair of Shaping Our Lives) attended all three groups.

All IIM group members identified as Disabled people and had been involved with Shaping Our Lives in various ways and degrees for several years, some occupying senior positions such as directors or officers. Recruitment to the group had taken place through an internal Shaping Our Lives process with members being invited to form the IIM project group once funding had been secured from the UK National Lottery. The main aim for the IIM group was to deliver the aims of this funding by co-designing two new services: the Involvement Champion (which later became the Inclusive Involvement Mentor programme) and the My Involvement Profile. Both new services were to be piloted in two sites in England once they were sufficiently developed.

The IIM group and the study group each met (independently) for two hours monthly, and both groups met for two hours at JIFs every 3 months. The data collected were audio-recorded transcripts and observational notes from online IIM meetings (26 h), online study group meetings (20 h), and online JIFs (8 h). BW also carried out semi-structured one-to-one interviews (44 h) with all project participants at two time points (month 6 and 10). To ensure confidentiality and anonymity, interview transcript data were not shared with IIM participants or study group members; only BW had access to this dataset which was used for purposes of inquiry and sense-checking but are not presented here as quotes could make interviewees identifiable (to others in the study).

As a collaborative endeavour and in the spirit of a participatory approach, how the data were to be analysed was decided collectively with everyone involved with the project consulted. Towards the end of the project we held several meetings to establish if and in what ways project members wanted to be involved in analysis and co-authoring of outputs. The options we discussed were (Table 2):

**Table 2:** Options for data analysis

1. A refinement of existing data analysis themes	BW used Thematic Analysis to analyse interviews, IIM and study team meetings and JIF transcripts, as well as observational notes, relevant documents such as Shaping Our Lives terms of reference and the National Lottery–funded proposal. Participants were invited to review the themes BW initially identified and to engage in two-way discussion to develop and refine these themes, e.g., by offering their views and constructive critique, and suggesting themes they felt may have been missed
2. Primary analysis of primary (non-confidential) data	Everyone involved was given the option to look at and analyse data in its pre-analysed state, e.g., group meeting transcripts, meeting minutes, relevant documents to identify themes without knowledge of the themes BW had separately identified
3. User-led group discussion	Members of the IIM group to share their own experiences of the collaboration with each other without engaging with any of the empirical datasets. IIM group members would discuss their experiences of the project and develop their own themes relating to the most significant and relevant factors and issues encountered during the project
4. Identify the main themes based on personal experience	Without reviewing the data or findings of the thematic analysis, participants were invited to share what they felt had been the most significant aspects of the project. This could include what they felt the main issues had been and what explained these, what had gone well, and why this was thought to have been the case?
5. Suggest an alternative analytical approach	It was appreciated that not everyone may want to engage with any of the previous options so everyone involved was invited to propose alternative ways that they could contribute to the analytical process

Our findings below are based on a synthesis of interpretations and understandings of options 1, 3 and 4 which were variously chosen by different members of the group.

## Results

### Getting underway: a bumpy start

Conversations regarding a collaboration between the research team and Shaping Our Lives started in February 2020 whilst Shaping Our Lives were awaiting the outcome of a National Lottery Community Fund (NLCF) grant proposal, that was eventually successful and became the IIM project. It was agreed between members of both organisations that the as-yet-to-be-formed IIM group might offer a case study through which to explore the potential utility of applying Ostrom’s principles within participatory practices. Consequently, we applied as joint-applicants to the Economic and Social Research Council (ESRC) for funding. In March 2020 Shaping Our Lives learned that the application for their NLCF grant had been successful. Seven Shaping Our Lives members subsequently came together to form the IIM group supported by a core member of staff, the head of projects.

Due to various setbacks, some relating to the COVID-19 pandemic, the IIM project did not get underway until October 1st, 2020. We found out that our research proposal to use Ostrom’s principles within this project was successful in November 2020. During this early period and prior to our study formally starting in January 2021, GR and OW held several meetings with the IIM group to introduce themselves and the aims of the research study. Initially, some IIM group members were aware of the planned study whilst others were not. Although senior members of Shaping Our Lives were joint applicants on the research funding application, some within the wider group who had not been involved at that earlier stage raised concerns about the level of influence a group of non-Disabled researchers might exert within a group of Disabled people, in a user-led context and there followed a series of conversations and e-mail communications relating to this.

The IIM group started holding monthly, two-hour meetings from January 2021 to begin designing services as part of their NLCF grant. The proposed services were the My Involvement Profile and Involvement Champion (later becoming the Inclusive Involvement Mentor programme). The My Involvement Profile was intended to form a document that would outline a public contributor’s or service user’s experience, skills, and the support and accessibility requirements they would need to take part in involvement activities for health and social care services. The My Involvement Profile was to be designed by the IIM group as part of formalising and improving inclusive involvement practice. The Involvement Champion was a new role for someone to work at the local level between user-led Disabled service user groups and organisations and local authorities (elected bodies that provide a range of services for a particular geographical area in the United Kingdom), also with the aim of improving and supporting service user voices as part of decision-making processes in accordance with Shaping Our Lives ethos of ‘nothing about us without us’.

Whilst staggered start times with the two funded projects may have played a part in the sense of a ‘bumpy start’, a larger issue perhaps lay in the lack of a sense of joint enterprise and clarity regarding aims at this stage. Agreement regarding the study had been reached between members of both groups (GR, OW, BM and PB) prior to the IIM group forming; potential members had been made aware of the collaboration when initially joining the IIM group but may not have had a clear understanding of its aims. Alongside questions of influence, there were also differences in the objectives of members of the collaborating parties that needed to be acknowledged. The IIM project was focused on co-designing services as part of the NLCF grant, it was task-orientated and had a clear remit, whilst the research study sought to engage with Ostrom’s principles in an exploratory and open-ended way. Whilst some IIM group members drew on their past experiences of collaborative and project working to minimise the ‘bumpy start’, the initial lack of clarity was nonetheless experienced by some as disruptive:

…there was agreement that there had been a period of confusion about roles, responsibilities, and the purpose of different meetings. For some the process of sorting this out had contributed to their understandings of co-production. For others the rectification process lacked involvement of the user group and re-enforced the power held by the academic team.

– feedback from IIM group, May 2022

The different language used by different members of the collaboration at this time highlights these divergent understandings. In the original study proposal, the language had been ‘adapting and applying’ the principles ‘to the task of co-designing the new services’. The research team thought they would be participating in a collaboration in which an exploration of how the principles could inform the work of the IIM project would occur:

Moving away from the tradition of ethnographers ‘delicately lurking’ to minimise their influence on what/who is being observed, this project will see a researcher join the IIM project team so that together we can reflect upon and observe the benefits and challenges of adapting and applying Ostrom’s design principles in this context.

– research proposal, case for support, June 2020

This project is the IIM project, and it is co-production and co-design that’s happening. The person we employ (researcher) will be involved working on the project not just an observer they will need understanding of the (IIM) Working Group. I don’t think the projects are separate as it’s a reflective project. We would like when starting the project in earnest to suggest that these principles might be used as guiding principles - some principles might work and others not and that’s what we can learn from and what can be also helpful to the project.

– extract from transcript of initial meeting between study group and IIM group, November 2020

However, rather than ‘adapting and applying’ the principles to the work of the IIM group as set out in the grant proposal, the language of ‘comparison’, ‘evaluation’ and ‘contrast’ emerged as an alternative framing of the research project during our early discussions:

This is a comparative evaluation looking at the eight principles Elinor Ostrom developed from studying community managed resources such as farmland etc. Elinor Ostrom developed one of the first models of co-production which draw on some of these principles. What we will be doing is comparing how a user-led working group from Shaping Our Lives uses certain methods and principles to achieve inclusive co-led projects. The methods and principles will be compared and contrasted with Elinor Ostrom’s eight principles to see where there are similarities and differences. It is not critically evaluating how we work. When this idea was created between [Shaping Our Lives] and King’s College London, we agreed it was a great opportunity to highlight the methods and principles of co-production in a user-led organisation; compared to an accepted academic set of principles used by communities to jointly manage community resources.

- discussion paper following Inclusive Involvement Movement working group, 25th January 2021

When clarity was sought regarding the aims of the study and what was meant by the ‘development of participatory research practice’, GR and OW explained that through the discrete task of co-designing the services with the IIM group as set out in the research grant proposal, new ways of working between academics and user-led groups might emerge. Some IIM group members queried if this ought to be the other way around i.e., establishing joint ways of working *before* attempting to ‘apply’ the principles, placing emphasis on getting to know one another initially. Whilst the original proposal had set out an attempt to ‘test and apply’ the principles, ongoing questions around the aims of the collaboration overall meant that there were questions and uncertainty about how this would work.

When the research team joined the IIM group in January 2021 there was a need to establish new working practices to incorporate the study into the wider working of the group. However, it proved difficult to agree on what these working practices should be. As the bridge between the two groups, it was intended that a researcher would work within and between both but due to delays in recruitment no one was in post yet. IIM group members expressed concern regarding their own time and capacity and their need to ‘get on with the job of co-designing the services’. In late January 2021 it was agreed that a separate advisory group would be established comprising those who co-wrote the original study proposal (GR, OW, BM and PB) and the researcher when recruited. This advisory group would convene monthly to discuss the IIM group’s work, the principles, and examine if, how, and in what ways they were being applied. The researcher was to document this process through participant observation and one-to-one interviews (at this point during the pandemic fieldwork was limited to online activities). Both the IIM and advisory groups would then come together quarterly throughout the 16-month project period in JIFs in which reflections would be shared:

It is important to stress; the advisory group do not have any authority over the [IIM] working group – they can merely advise and contribute their expertise when it deems appropriate, but this advice will be given in the full knowledge that the working group can decide to do things as they see fit. Ostrom’s principles are not being put forward as a way that things must be done – they are just something to reflect with and may or may not be deemed useful by the working group … Together we reflect on what impact, if any, Ostrom’s principles have over the course of the project (and if so why). What seems important to emphasise at this point is that the working group have a job to do – developing the Inclusive Involvement Movement project work – and the [research study] should not be a distraction to this!

- answers and responses from research team to a discussion paper following Inclusive Involvement Movement working group, 25th January 2021

However, the research team and IIM group continued to have different ideas about how the research project was going to meet its aims. Whilst the research team felt that the most realistic way to 'adapt and implement' the principles in the lottery funded project was through discussing the potential relevance of the principles in contextually relevant ways and at times when it was agreeable to the wider group (fitting in with them rather than driving an external agenda), the IIM group tended to see the two projects as rather more separate:

[The research study] is not influencing the Inclusive Involvement Movement project just looking at academic side, not advising on the work but on the principles. The advisory group will reflect if using a principle might be helpful to the Inclusive Involvement Movement working group. Communication is intended to be both ways not a hierarchal structure … The Inclusive Involvement Movement Working Group aren’t using Ostrom they are being observed what principles they are using, and this is to be compared and contrasted with [the collaborative group working principles].

- study group minutes, 25th March 2021

With an emerging agreement to keep the principles at arm’s length from the IIM group, when the researcher joined the team in March 2021, she (BW) was asked to produce a document outlining her understanding of how the IIM and research projects were related and would be undertaken going forward. In subsequent meetings, IIM group members expressed their feeling that the principles were complicated and requested they be put into plain English. It was acknowledged that as experts by experience the IIM group wanted to undertake the process of translatio’ into their own user-led context. This was an opt-in activity, and some IIM group members engaged with this process and attempted to interpret and re-write the principles whilst others chose not to. At this stage, it became clear that the proposed application of Ostrom’s principles directly within the IIM group’s work was not going to be as straightforward as it was initially outlined in the original, co-written research funding proposal.

### Impasse and finding our way

Concerns about the influence of non-Disabled researchers, and the relevance of the work of Ostrom (also a non-Disabled academic) meant that the study was now taking place one step removed from the IIM group activities. There continued to be a felt lack of clarity regarding aims:

Is the IIM working group working independently and being observed? Understood we should be looking at the processes and not the decisions made. People shouldn’t be influenced how they make decisions. Why was the Joint Interpretive Forum looking at the problems in the project? We told the [researchers] observe and don’t take part. If that’s altering [we] need to go back to them and get agreement.

- advisory group minutes, 14th October 2021

It became clear that for some IIM members ‘user-led’ meant the IIM group must work completely independently from the researchers and the study. The notion that IIM members should not be influenced in how they make decisions raised pertinent questions regarding the feasibility of the original aims of the study as laid out in the co-written application, as well as highlighting that ‘user-led’ is variously defined and understood. Some IIM group members felt that there should be no influence from researchers who were not Disabled and not contributing experience primarily gained as a service user and that the IIM services should be co-designed solely by people with lived experience. Other IIM group members felt that influence and ideas from others were not problematic and collaborating with them did not contradict a user-led approach because ultimately they (the service users) would be making the decisions:

*IIM member A*: …my expectations coming in, that this will be collaborative, and for me, collaborative doesn't necessarily mean that the King's College will be influencing user-led organisation. To me, it meant that they will bring knowledge and help us understand the vast amount of knowledge that they could have potentially contributed.

*IIM member B*: Isn't that the same thing? Isn't bringing knowledge influencing in some way?

*IIM member A*: It's up to us to interpret that knowledge and decide what you do, as user-led. It doesn't necessarily mean that that is what you will do, for me, anyway. That's how I would have -- there is an information and knowledge gap that is potentially real. But the point I wanted to make is you couldn't have done that in the beginning because you just didn't know that this was going to happen, particularly because we, as a user-led organisation, as the intent that we will always remain in control.

*IIM member B*: I kind of disagree on some of the points you made. I personally didn't have a problem with King's bringing knowledge or even King's influencing, in the same way that I don't have a problem with Shaping Our Lives bringing knowledge and Shaping Our Lives influencing. It's what happens when you work with different organisations, isn't it?

*IIM member A*: Yeah. I'm just reflecting what I felt that happened, particularly that September joint forum meeting. It was very clear for me that there was a -- whether it's conflict, confusion, etcetera

- IIM members, group discussion April 2022.

The sense of the importance of *our own knowledge* is at the heart of Shaping Our Lives’ philosophy. In this context the request for a comparative and evaluative piece of work, running parallel but not influencing the IIM group, was seen as desirable in enabling a greater understanding and promotion of Shaping Our Lives’ own user-led approach:

When this [the research-funded study] idea was created … we agreed it was a great opportunity to highlight the methods and principles of co-production in a user-led organisation; compared to an accepted academic set of principles used by communities to jointly manage community resources. This idea was fuelled by the fact that some of Elinor Ostrom’s principles are similar to the ways a user-led organisation work. Other principles may differ and that is fine. The researcher … will help to identify opportunities when Ostrom’s principles may be relevant to an approach we have taken or raise a principle that may not have been used. We do not have to follow Ostrom’s principles, and there is no measurement of success against them. It is a comparison of approaches and at the end we will have a documented study of user-led working, which will hopefully be given more credibility and acceptance because it has been studied alongside some Nobel prize winning work!!

– discussion paper following IIM working group meeting, 25th January 2021

The wish of some members of the IIM group was to illustrate Shaping Our Lives existing user-led practice through demonstrating how it compared to Ostrom’s non-Disabled academically-orientated work; this emerged as an important difference in understandings between Shaping Our Lives and the research team.

There was definitely tension, and I recall, and I can't remember which meeting it was, but it was a joint forum, where there definitely was a tension about the role of the university here, in what we call the collaboration with the IIM group. There was a feeling that it was becoming a bit too engaged in the task rather than the exploration. And that didn't seem appropriate. It certainly didn't feel to be appropriate, if I'm honest.

– IIM member, group discussion, April 2022

As researchers interested in participatory research, the researchers felt they could work equitably with the IIM group and that together they were embarking on a joint venture that explored the potential utility of an innovative methodological development. The researchers saw the principles as having potential to support user-led and other organisations in the co-design of sustainable services and potentially aiding the development of collaborative working practices. However, at times the researchers felt that they were being positioned as academic outsiders in a stereotypical us and them divide. When an IIM member expressed the sense of being ‘done to’, this was precisely what the researchers had been keen to avoid. Further questions of power relations were raised:

User-led research is associated with three principles: equalising the relationship between the researcher and the researched; working for the empowerment of the research participants; and purpose of the research is for making change in the interest of the people being researched. Possibly any of us could be criticised for not paying attention to equalising the relationship between service users, Disabled people, and the researchers – assuming we’d be doing that and not recognising everyone isn’t used to doing that.

– IIM meeting minutes, 25th October 2021

As described above, the IIM group discussed the research project in relation to user-led research where the dynamic is one of academic researchers working for the empowerment of research participants. However, it is debatable whether this is what the collaboration was setting out to do, as the original co-written research proposal states:

Our project brings together social scientists from King's College London and Shaping Our Lives - a national organisation led by and for Disabled people - to achieve two aims: (1) to work in partnership to develop new services that will support Disabled people and service users to become more involved in research and (2) to test a new approach to partnership working to help researchers and service users work together more fairly and effectively.

– research proposal, case for support, June 2020

The emphasis in the proposal was on partnership working, not user-led research per se. It is important to acknowledge this difference as it relates to questions of power, the purpose of academic research, particularly collaborative research, and commitments to critical inquiry. Informed perhaps by differing professional, discipline specific, and sectoral backgrounds with associated differing theoretical and philosophical understandings, there hence emerged different understandings and expectations of what the collaboration was about and how partnership was to be appropriately practiced. Time was needed to develop joint understanding.

It was in this context that in October 2021 it was decided to attempt to address the issues outlined above. Agreeing that it would be necessary to use a clear methodological approach to offer structure in a complex context, a proposal was developed suggesting that, based on the observations of IIM group working, the researchers would choose an aspect of the IIM group’s work to apply the principles to and to explore this in subsequent discussion at the next JIF. This shifted the focus away from the sense that the principles were being applied to the IIM group itself and how they were working as a group and instead to a specific element of work the group were undertaking. This was usually an aspect of their work that was still in progress and different views on how to progress with it had been expressed within the group. The proposal was that BW, OW, and GR would develop a briefing paper outlining the suggested application, i.e., the ways in which the principles might be relevant or useful in relation to an element of the IIM groups work, and then for this to be shared with the IIM group ahead of the JIF where it would be discussed. In some regards the adjustment could be argued to have run counter to what it appeared some members of the IIM group wanted in seeking to minimise input from the researchers. However, as a jointly developed response, this proposal was received positively by IIM members and was considered in the spirt of adaptation and flexibility that had originally been proposed as part of the collaboration.

### Influencing practice—shifting from shared resources to shared endeavour

The September JIF had bought to the fore a key issue; the level of influence of researchers who were not Disabled nor contributing experience primarily gained as a service user might have in working with a Disabled, service user-led group. While some IIM members had no issue with the study and drawing on the work of a non-Disabled academic in Ostrom, others did, and this needed to be addressed. In seeking to agree a new approach on which the IIM group reached some consensus, we had at least found the basis for a way forward and the December 2021 and March 2022 JIFs enabled a more in-depth and applied discussion of the IIM project work in relation to the principles.

Building upon this shift, another crucial step forward was the shift away from the interpretation of the ‘boundaries of the shared resource’ described in Principle 1 as the ‘extent of the IIM group members experience and knowledge’ which seems to have initially been the case for some. Instead, we agreed it might be more helpful to shift the focus from thinking about the ‘boundaries of the shared resource’ and instead think about the ‘boundaries of the shared endeavour’, i.e. what were the parameters of the task—of co-designing the services. This helped to provoke a clear delineation between the ways of working of the IIM group and the task of co-designing the services and was viewed as a helpful and important development:

I think the mistake we made initially was in wrongly identifying the resource. Looking back on it, if we looked on the resource as being the group's experience and knowledge, that, if you like, I think led us to feel or could have led people to feel that their experience and knowledge was being challenged by the principles. And I think that may have been at the root of some of the initial misunderstandings. Once we got the resource right as being the actual job, you see what I mean, I think that was a much more comfortable position because principles could be applied to the job and not to the people in the group. And so, I think perhaps the initial mistake we made was in wrongly identifying what the resource, if you like, that we were managing that might be the subject of the principles, really was.

– IIM member, group discussion, April 2022

And I think one of the things we found here was that there are two, actually quite disparate aims that we're trying to marry together. And the two disparate aims are one, looking at the applicability of Ostrom's principles to something that is not a tangible resource, and a way of working that is not necessarily the same as a group working to manage that tangible resource on the one hand. And secondly, the actual creation of the resource. In other words, there isn't a resource that we're managing. What we're doing is creating something which is the resource or the task. And I think, had we understood that from the beginning, and had we done more work in marrying the two together, some of the language difficulties may have been overcome.

– IIM member, group discussion, April 2022

The helpfulness in this shift in interpretation is illustrated in how the groups subsequently explored the relevance of the principles in relation to the task of co-designing a mentor role as part of the NLCF-funded project (as discussed below).

#### Applying the eight principles to the Involvement Mentor role

In this section we review how exploration of the potential relevance and utility of each principle in specific relation to the Involvement Mentor role (something we did in the JIFs) facilitated the development of this element of the IIM project. These findings are summarised in Table 3.

**Table 3:** Relevance of principles to Involvement Mentor role

Principle	As applied to Involvement Mentor role
1. Clearly defined boundaries	Facilitated thinking about service design by highlighting differences between (a) a mentor-in-training as someone who might work with the IIM group, versus (b) a fully trained mentor working independently of the IIM group
2. Proportional equivalence between benefits and costs	Enabled a consideration of how the mentor role might vary regarding payment and reward
3.Collective-choice arrangements	Not seen as relevant given the IIM group felt they already made their own rules and decisions
4. Collective endeavours are inherently vulnerable to ‘free-riding’ and active exploitation	Facilitated thinking about the need for the IIM group to retain a degree of control over the co-designed services, to avoid external groups or individuals realising benefits without contributing to the wider project
5. Graduated sanctions	Enabled the IIM group to think about how they would respond to scenarios where, for example, a mentor was not performing their role in the anticipated way or how sanctions would be applied if a mentor was employed by another organisation
6. Conflict resolution mechanisms	Enabled a discussion of what might happen if conflict arose between Shaping Our Lives and an organisation hosting a mentor
7. Minimal recognition of rights to organize	Raised questions of self-governance, the external constraints that the co-designed services would operate within, and the need for the IIM group and Shaping Our Lives to be able to determine the nature of their relationships with other organisations (including whether a contractual approach was feasible or desirable)
8. For groups that are part of larger social systems, there must be appropriate coordination among relevant groups	Relevant to IIM group and Shaping Our Lives as they were attempting to co-ordinate activities with other groups and organisations. Meaning of ‘every sphere of activity has an optimal scale’ was challenging to apply to the IIM group’s work

Some IIM members felt exploring the relevance of Principle 1 (clearly defined boundaries between the resource [/task] and group) enabled them to think about the design of a mentor role which was something that had developed out of the co-design process. It helped to make explicit and consider the differences between (a) a mentor-in-training as someone who might work with the IIM group, versus (b) a fully trained mentor working independently of the IIM group. IIM group members highlighted the fixed lifespan and project-based nature of the IIM group and felt that mentors could not feasibly be part of a group that was to be disbanded, whilst also acknowledging that feedback mechanisms to Shaping Our Lives were necessary to enable learning, development, and alignment with Shaping Our Lives intended ways of working. As such, our new approach to considering the principles facilitated thinking about service design in ways that had not fully-developed in the co-design process prior to this discussion.

As originally interpreted, discussion of Principle 2 (proportional equivalence between benefits and costs) had focused on challenging questions regarding ‘high status and disproportionate benefits being earned’. Group members discussed how Disabled people do not always have the same ability as non-Disabled people to earn and achieve high status and as such some felt this principle was unfair and not in keeping with Shaping Our Lives ways of working. In contrast, when focus had shifted to thinking about the shared endeavour or task of the co-design of services, discussion of Principle 2 enabled a consideration of how the mentor role might vary regarding payment and reward and challenged a one-size-fits all approach to value:

…this principle is relevant to the future; some mentors will be volunteers and others employed by an organisation wanting inclusive involvement. Issue of proportionality, as some might get paid, and others won’t. Doesn’t think Shaping Our Lives will be in a position to employ them, other than possible involvement payment during training. Will need to address that some mentors will be better off financially than others. Organisation’s principles may not align with Shaping Our Lives and some organisation may want more involvement than others, don’t think Shaping Our Lives can control that.

- IIM member, Joint Interpretive Forum meeting minutes, 6^th^ December 2021

The recognition that (as stated in principle 2) ‘unfair inequality poisons collective efforts’ was useful as a way of understanding variation in status, with reward not being detrimental if it is collectively agreed upon. This raised further questions of incentivisation:

It becomes important in the pilot and how we build it. If the mentor is a role and that is to increase meaningful involvement, need to find way to incentivise them. The more meaningful involvement happens when there is some financial reward.

- IIM member, Joint Interpretive Forum meeting minutes, 6^th^ December 2021

Questions of recognition and reward relating to a chosen pilot area as discussed in the subsequent March JIF were subsequently incorporated into the mentor role:

What is in it for the mentor? The mentor will take part in this programme of activities as a volunteer. However, Shaping Our Lives will provide rewards in the form of training, peer support, networking, skill development, reach to other involvement work and a recognised status as someone who has completed our mentor programme.

- about the mentor pilot in a University, March 2022

Principle 3 (collective choice arrangements) was considered irrelevant to the co-design of the mentor role as the IIM group felt they already made their own rules and decisions. Group members discussed how trust and friendship were important aspects of their working and enabled open dialogue that avoided the necessity for more formal measures, though those were there if necessary. Initially the group did not feel that Principle 4 and questions of freeriding and exploitation were relevant either as unlike with CPR management there was no material resource to exploit and group members were motivated by a desire to contribute without an expectation of benefit. Again, however, when considering the relevance of the principles in relation to the shared endeavour of co-designing the mentor role it was possible to see the ways in which Principle 4 might be useful. Applied to organisations or individuals who might utilise a Shaping Our Lives mentor for their own ends, Principle 4 appeared to facilitate the group to think about the need to have a degree of control and avoid ‘people taking benefits but not delivering’ (JIF, 6th December 2021):

This principle becomes very relevant further down the line. If you employ someone then can deal with freeriding under the contract, more problematic if they are volunteer or employed by someone else.

- JIF minutes, 6^th^ December 2021

Principle 5 (graduated sanctions) and questions of transgressions were similarly felt to not be relevant to the IIM group itself, but when applied to the role of the mentor it was again found useful. As was highlighted, ‘… if they [a mentor] become part of the group these sanctions would also apply to them, but how is that affected if someone else employs them?’ (JIF, 6th December 2021). Would the mentor be contractually obliged to operate within the rules of a host organisation? What might happen if Shaping Our Lives felt the mentor was not carrying out the role in the expected way? The issue was raised again in the final March 2022 JIF regarding the pilot areas as testing sites for the mentor service:

How can Shaping Our Lives impose rules on another external organisation – needs thinking about. What do we do if we want to work in a certain way and want mentor to work in certain way and their organisation don’t want to?

- IIM member, JIF minutes, 14th March 2022

When discussing the pilot areas in the March 2022 JIF, Principle 6 (conflict resolution mechanisms) enabled a discussion of what might happen if conflict arose between Shaping Our Lives and a host organisation:

Members have previously stated that there is already an agreed system in place for this within the Inclusive Involvement Movement group. Are there any aspects or issues relating to the pilots specifically which might require revisiting this, such as volunteer policies for mentors that could draw on the existing systems?

– JIF briefing paper, 14th March 2022

Applied to the mentor role and pilot areas, Principle 7 (minimal recognition of rights to organise) raised questions of self-governance, the external constraints that the co-designed services would operate within, and the need for the IIM group and Shaping Our Lives to be able to determine the nature of their relationships with other organisations. The group discussed whether a contractual approach was feasible or desirable. It was suggested that such formalisation of relationships might not always be desirable, highlighting instead a preference for relationships based on trust in which potential disagreements could be managed without recourse to formal processes. It was discussed that there might be a ‘run-in’ period prior to any partnership work starting. During this period agreements would be made in the form of a ‘memorandum of understanding’, while mentors were familiarised with the host organisation and vice versa and ‘softer’ forms of agreement might be preferable.

In that the IIM group and Shaping Our Lives were attempting to co-ordinate activities with other groups and organisations, it was clear that Principle 8 (appropriate coordination within larger systems) had relevance to their work. While the meaning of ‘every sphere of activity has an optimal scale’ was perhaps challenging to apply to the IIM group’s work, it seemed to make enough sense for it to be considered relevant in this context:

I thought Principle 8 related to Shaping Our Lives and it’s wider 400-member network as well. We want to enable people to play a much bigger role in determining the quality of their lives, getting services that really meet their needs, and we make that more available by the efforts we’re making now. So, I see those principles as well connected.

– IIM group member, Inclusive Involvement Movement group minutes 26th April 2021

Using the principles to discuss specific aspects of the co-design work helped to (a) provide a way for the two groups to explore the relevance of the principles in a productive rather than divisive way and (b) helped develop thinking on specific elements of the project which supported planning and actually doing the co-designing of the new services.

#### Reflections

When asked whether the principles had played a part in decision making over the 16-month period, individual IIM group members gave varied responses:

Where we went through taking each principle and looking through the lenses of those principles, particularly the areas of mentor and the pilot and setting it up. And I recall actually going through each principle and examining it through the lenses of the principles. I thought that was a wonderful way of actually adapting and using those principles. So, I found that really, really valuable because actually it examined the applicability of what we were doing. And we had examined all the possibilities.

– IIM member, group discussion 27^th^ April 2022

To what extent they actually influenced me in the discussion I entered into in helping determine what we adopted, I'm not sure, but I suppose in truth, I never at the time, when we're entering a discussion about what we were going to decide upon, that I was thinking, ‘that meets that principle or that meets that principle’. So in that sense, they weren't playing a part. But I was always aware of them, because we were cojoined in a way, I made myself aware of them. To what extent they influenced, I can't be sure.

– IIM member, group discussion 27th April 2022

The first quote above illustrates how shifting to applying the principles to a specific element of the groups working and shifting from thinking of the shared resource as the knowledge and skills of the group to being the shared endeavour of co-designing the services enabled a new and useful facilitation device for some IIM members. It is possible to consider these shifts perhaps as forms of stepping back from an interpretation of the principles for collaborative group working that saw us focus on the *group* itself and how they were working and instead thinking more about what the group were *working* on. Others were more critical and saw little to no value in attempting to work collaboratively with the principles as we had attempted:

Exploring the principles was not needed as Shaping Our Lives does this already.

– feedback from Shaping Our Lives project group, May 2022.

This criticism is illustrative of a wider theme too. Although members differed in terms of how problematic they felt this to be, a number of members of the IIM group felt that the principles were already present within the group prior to and without their introduction:

When I look at those eight principles, I think any community-based organisation that is successful must operate within them. And I had no difficulty with the [concept of] resource. And I always thought knowledge was the resource…I just feel they're a very good set of principles for any community-based organisation that is successful to operate with.

- IIM member, group discussion 27th April 2022

For me, I just know that it's good practice to have a volunteer agreement from working in the voluntary sector for decades, this is what you do. It's so that everybody knows where they stand, so that the boundaries are clear, etcetera. And yes, those things I just said may well be in the principles, but they're also elsewhere. And I know it's good practice, etcetera. I find it hard to say, ‘Yes, it was definitely due to the principles’. Who knows? I think it's hard for anybody to say, isn't it?

– IIM member, group discussion 27th April 2022

Given that Ostrom’s work was rooted in empirical investigation it is perhaps unsurprising that there would be pre-existing aspects within a group. As discussed earlier, some of the principles were not felt to be adding anything novel as the group were already doing something similar. It is interesting to note where the principles were not pre-existing and where with some adjustment to the methodological premise of the research project they were felt to be useful to draw upon. Nonetheless, the IIM group acknowledged there was variation within the group regarding how helpful the principles were and how successful the wider collaboration had been:

Views on most points discussed were on a spectrum from negative to very positive. There were sometimes as many different perspectives as participants. Questions were raised and pondered, but conclusions not always reached. However, the group felt able to disagree and express their different opinions, a reflection of the views expressed by some that a clear benefit has been getting to develop a good working relationship with colleagues.

– feedback from Shaping Our Lives project group, May 2022

There was agreement that a longer familiarisation period or ‘phase zero’ [25] would have been beneficial:

It was an ambitious project which was made more difficult by the pandemic, the work demands of the lottery project in the first few months and delay to recruitment of a research associate…although planned, in practice there wasn’t the capacity in the one meeting to discuss this project as well as the actual lottery funded one…more meetings between the IIM group and research team at the start of the project would have been beneficial…there was a consensus that more time at the start of the project working out how to work together would have been beneficial. It was acknowledged that remote working [not originally planned but forced upon us by the COVID-19 pandemic] had not allowed time for the downtime of the ‘coffee and fag break’ of in real life meetings which are often ice-breakers…

– feedback from Shaping Our Lives project group, May 2022

And I think the groundwork, if you like -- understandably, in many ways and I think this may have been a result, partly, of having to meet remotely all the time, but some of the groundwork in terms of where the projects meshed together, what you were trying to achieve, what the resource was and how to put together the different aims of the project, I think that was missing. And I think we only started to do it as the two projects proceeded and possibly only got it right by about October when we, as you say, identified the resource and altered the methodology.

– IIM member, group discussion, April 2022

The second aim of the collaboration was to test a new approach to partnership working inspired by Ostrom’s principles for collaborative working that could help researchers and service users work together more fairly and effectively. There was a lack of clarity surrounding whether Ostrom’s principles were being directly applied to the wider collaboration as opposed to just the IIM group, which was experienced by some as confusing and problematic:

The impact of the changing goalposts [reference to deviating from the aims set out in the application for ESRC funding] is not fully reflected. I was hoping to explore the relationships between academic departments and DPULOs [Disabled People’s User Led Organisations]. This did not happen.

– feedback from Shaping Our Lives project group, May 2022

Despite challenges, a majority of IIM group members experienced the project positively overall and the collaboration as constructive:

Previously thought [Shaping Our Lives members] had lesser status when interacting with academics but in this project felt recognised and valued, so will now approach other university partnerships with the confidence of being an equal partner.

– feedback from Shaping Our Lives project group, May 2022

## Discussion

The principles were not applied in the way expected and as originally proposed in the research grant application. The first half of the project involved various hurdles and disagreements that were experienced as difficult and time consuming to resolve by most of the collaboration members. However, as the partnership developed and relationships between the collaborators grew, adaptations in our approach were made and a rethinking of what the principles for collaborative group working were being applied to enabled us to overcome these obstacles; we were then able to examine the relationships between the principles and the IIM group’s work more closely. Whilst there may have been some influence on the IIMs co-design of the services—particularly in the latter stages when the principles were found to be useful—we consider that nonetheless the principles were used predominantly as a facilitation device, providing a theoretical sounding board with which to think through and discuss various scenarios and possibilities relating to the co-design of the services. In applying the principles in a more rigorous way and to the task as opposed to the group itself the principles were re-positioned and this speaks to their malleability and potential utility. In continuing to work together and adjusting the approach we strengthened the collaboration, arguably meeting objectives relating to our second research question of whether a new approach to partnership working could facilitate researchers and service users to work together more fairly and effectively. It should be noted that some of the initial communication difficulties experienced in this project are at least partly attributable to the severe limitations the COVID-19 pandemic forced us to work within. Being forced to work entirely online with groups who have an expressed preference (need even) for fostering partnerships and working in collaboration in person certainly did not help the establishment of shared understanding and good working relations.

We might also ask what a delineation between the group and the task in relation to the interpretation of Principle 1 means and why it was experienced positively and enabled the project to move forward. In this project it helped to quell the sense/fear that the researchers were judging (from a position of superiority) the competency of the IIM group and instead facilitated discussion of the *possible* (rather than presumed) relevance and usefulness of the principles to elements of what the group were working on. The discussion was based on *offering* the principles as potentially relevant and useful to the task of co-designing the new services rather than as standards of quality the researchers were judging the group against (which was never the intention, but the project was interpreted by some in this way). Ostrom built adaptability into the principles so that they could be applied in a variety of contexts and highlighted the need for auxiliary principles to make them applicable therein [16]. Our experience of attempting to apply them in this project reflects the potential usefulness of their malleability and emphasises the helpfulness of responding to the specificity of any given collaboration to promote effective and fair working relations. Further work is seeking to address this need by re-classifying Ostrom’s principles in ways that relate them to three distinct aspects of co-design [26]

Confusion had also been bought about by the wording of Principle 1, and there was some agreement among collaborators that we got ‘stuck’ at the stage of interpretation and adaptation. In earlier discussions, IIM group members had queried how there could possibly be a delineation between the identity of the group as Disabled service users and the resource as the knowledge and skills of the group. It is clear to see why this would have been experienced as problematic. As a user-led organisation of Disabled service users adhering to a philosophy of nothing about us without us, it was fundamental that the co-designing of the services was led by people with relevant lived experience. The identity of the group was fundamental to the resource. It is possible that for Ostrom identity meant something different, more prosaically who is in and who is outside of the group, rather than being applied to a group of people for whom identity is an important philosophical concept (raising questions regarding interpretation, translation, and applicability of Ostrom’s principles in user-led contexts). However, it was in the nature of academic study that our starting point was the work of Ostrom and then we proposed exploring what Shaping Our Lives did in relation to that; we might equally have started with the ways of working of Shaping Our Lives and then explored Ostrom from their perspective, a suggestion which was made early on by some members of Shaping Our Lives.

Such questions surrounding the power and influence of researchers (including Ostrom) became particularly pertinent. When taken into the wider IIM project group, misunderstandings, and differences of opinion between parts of the collaboration were illustrated through different uses of language (‘compare and contrast’ as opposed to ‘adapt and apply’ and ‘in partnership’ as opposed to ‘user-led’ for example). In the final months of the project, from December 2021 onwards, there was a greater sense of collaboration and through this developed a more considered approach to the principles. Suffice to say, the principles might be considered as having facilitated discussion that supported co-designing the IIM services and to some extent can be thought of as prospectively informing the groups work. Certainly, it can be argued that through lengthy, in-depth discussions ideas were considered that might not have otherwise. Although of course we cannot be certain that the end result would have been different if the principles were not considered. The reflexive space of the JIFs supported the group’s thinking about how the principles might apply in ways that were felt by some members to be practically useful.

The period prior to October 2021 felt like a process of various unsuccessful attempts to ascertain what applying the principles meant in practice. These attempts were unsuccessful because a) the partners were yet to develop the working relationship and trust that is often a prerequisite for productive use of innovative approaches b) not all members of the collaboration co-wrote the research funding application (as the IIM group was formed after it was submitted) and so were not necessarily onboard with the premise of the collaboration in the first instance or took part in early attempts at collaboration, and c) there was not enough familiarity with the principles i.e., it took time to get to know and understand (and so work with) them. The researchers and members of Shaping Our Lives were aware of the likely usefulness of a ‘phase zero’ [25] (where all team members could get to know one another more and develop shared aims and understandings for a project). However, the difficulties around developing a shared understanding of what the project was largely stemmed from different funding timeframes meaning that one project started before the other was funded and then the pandemic made it impossible for us all to meet once funding had been secured for the research study (a situation we could not have predicted when applying). Although the research grant application was co-written, this was between senior members of Shaping Our Lives team and researchers at King’s College London. The IIM group found out only after funding was secured and some understandably felt as if it was sprung on them, that it had not been very collaborative, and that it could potentially get in the way of delivering on the aims of the IIM project. There are certainly lessons that could be learned about how and when to communicate the possibility and uncertainty of collaborations that have applied for and are reliant on external funding, especially if the collaborations are intended to engage with and influence existing projects. The lack of communication about the prospect of the study informing the IIM project at some stage seemed key to spawning different interpretations of what the study was and how it was to be conducted, and at times the perception that there was an us and them.

The project also raises questions regarding the ways in which ULOs approach and manage forms of other or outsider knowledge and how or whether collaborations with researchers can occur within user-led approaches. As far as we are aware, Ostrom does not address the question of outsider knowledge in the management of resources. Indeed, it is the knowledge of local people as experts that is explicitly valued in Ostrom’s work. What became clear in this project was that in the context of collaborations between academic researchers and ULOs it cannot be assumed that there is a shared understanding of what working in partnership or taking a user-led approach means in theory or practice, and that differing understandings can lead to confusion and tension. A key learning from this is that early on in collaborations, groups using such terms/approaches should openly discuss how each member defines and understands relevant methodological terms and reach agreement as to (a) the nature of the planned collaboration and (b) what terms people in the group feel comfortable using to describe this approach.

## Conclusions

Our collaboration was proposed as an explorative study so to some extent the unexpected is to be expected and embraced. The eventual approach we settled upon enabled the consideration of the principles in ways that appear to have been experienced as useful by most participants and enabled a way of working together that met the aims of the collaboration, albeit in unanticipated ways. The principles played to their somewhat esoteric language and proved adaptable to a range of contexts, as Ostrom had suggested. A conceptual shift from focusing on the resource as the knowledge and experience of the IIM group to the shared endeavour of co-designing the services instead was significant, enabling new ways of thinking about the group’s task. Overall, our study reinforced the importance of being reflexive and responsive when seeking to apply Ostrom’s principles for collaborative group working but also identified that if their potential utility is to be realised there is a clear need for the principles to be adapted and translated in ways that make them more accessible, contextually applicable, and usable by groups who do not have prior knowledge of Ostrom’s work.

We have published an accessible report [27] and a blog series [28] which shares learnings and reflections about partnership working between a ULO and academic researchers as derived from this project. The report focuses on the challenges of this type of partnership and how to capitalise on the opportunity of bringing a user-led organisation and university research team together to complete a research study. There is much to be learned about the theorising and methodologies developed by Disabled people’s and other lived experience movements. This learning is equally important in promoting more and better collaboration between ULOs and academic researchers.

## Data Availability

The datasets generated and/or analysed during the current study are not publicly available due to reasons of individual confidentiality.
